# Key Enzymes in Fatty Acid Synthesis Pathway for Bioactive Lipids Biosynthesis

**DOI:** 10.3389/fnut.2022.851402

**Published:** 2022-02-23

**Authors:** Xiao-Yan Zhuang, Yong-Hui Zhang, An-Feng Xiao, Ai-Hui Zhang, Bai-Shan Fang

**Affiliations:** ^1^College of Food and Biological Engineering, Jimei University, Xiamen, China; ^2^Department of Chemical and Biochemical Engineering, College of Chemistry and Chemical Engineering, Xiamen University, Xiamen, China

**Keywords:** bioactive lipids, desaturase, elongase, fatty acid synthesis pathway, oleogenic microorganisms

## Abstract

Dietary bioactive lipids, one of the three primary nutrients, is not only essential for growth and provides nutrients and energy for life's activities but can also help to guard against disease, such as Alzheimer's and cardiovascular diseases, which further strengthen the immune system and maintain many body functions. Many microorganisms, such as yeast, algae, and marine fungi, have been widely developed for dietary bioactive lipids production. These biosynthetic processes were not limited by the climate and ground, which are also responsible for superiority of shorter periods and high conversion rate. However, the production process was also exposed to the challenges of low stability, concentration, and productivity, which was derived from the limited knowledge about the critical enzyme in the metabolic pathway. Fortunately, the development of enzymatic research methods provides powerful tools to understand the catalytic process, including site-specific mutagenesis, protein dynamic simulation, and metabolic engineering technology. Thus, we review the characteristics of critical desaturase and elongase involved in the fatty acids' synthesis metabolic pathway, which aims to not only provide extensive data for enzyme rational design and modification but also provides a more profound and comprehensive understanding of the dietary bioactive lipids' synthetic process.

## Introduction

Lipid plays a critical role in maintaining the normal function of growth and metabolism, which is not only an essential factor for the fluidity of the plasma membrane but is also a carrier to store material and energy (1). Long chain polyunsaturated fatty acids (LCPUFAs) are the main active and functional components in lipid. With the continuous improvement of people's life quality, more and more attention is paid to the intake and proportion of various kinds of LCPUFAs in the daily diet (2). The fatty acids in the daily diet are mainly consisted of saturated ones and unsaturated ones. According to the different positions of unsaturated double bonds, LCPUFAs can be mainly divided into ω3 and ω6 series, such as docosahexaenoic acid (DHA, C22:6^Δ4,7,10,13,16,19^ ω3), eicosapentaenoic acid (EPA, C20:4^Δ5,8,11,14,17^, ω3), and arachidonic acid (AA, C20:4^Δ5,8,11,14^, ω6) (3). These LCPUFAs also play a crucial role in not only growth and brain development but also in preventing cardiovascular diseases, hypertension, and diabetes (4). Therefore, the Food and Agriculture Organization of the United Nations (FAO) and the World Health Organization (WHO) issued a joint statement that the intake of LCPUFAs fatty acids daily should not be <1.3 g (5). However, mammals can't *de novo* synthesize these LCPUFAs, which make getting supplements from diet to be particularly important (6).

In daily diet, the intake of LCPUFAs is the primary driver of deep-sea fish and vegetable oil. Adapt to a low-temperature marine environment, deep-sea fish oil is rich in LCPUFAs, enriched from the marine microalgae (7, 8). Some plants such as soybeans, flax, and peanut, can store lipid in their seed, from which humans could extract and harvest linoleic acid (LA, 18:2^Δ9,12^, ω6) and α-linolenic acid (ALA, C18:3^Δ9,12,15^, ω3) rich oil (9, 10). However, harvesting the LCPUFAs from plants and deep-sea fish is a time-consuming process, which was also limited by climate, land and ecological environment (11, 12). Furthermore, the plant could not provide LCPUFAs of DHA and ARA, while overfishing caused more deadly consequences, including destructive fishing practices, killing off the fish, and breaking the ecological balance (13). Thus, with the explosion of population, the LCPUFAs harvest speed from these two approaches can no longer meet market demand needs. Fortunately, the development of LCPUFAs from oleaginous microorganisms and microalgae provides an alternative source for increasing market demand. At present, many LCPUFAs' production processes have been developed, including *Schizochytrium* sp., *Mortierella alpina, Thraustochytrids* and *Yarrowia lipolytica* (14–17). Compared with LCPUFAs from plant and fish oil, the LCPUFAs driver from microorganisms and microalgae own many advantages. On the one hand, it broke the loose of climate and ground; on the other hand, higher proliferation, growth and LCPUFAs rich oil accumulation rate enhance the productivity and low the cost. Thus, the development LCPUFAs rich oil strategies attract more attention from scientists worldwide, promoting the development and research rate significantly.

**Figure 1:**
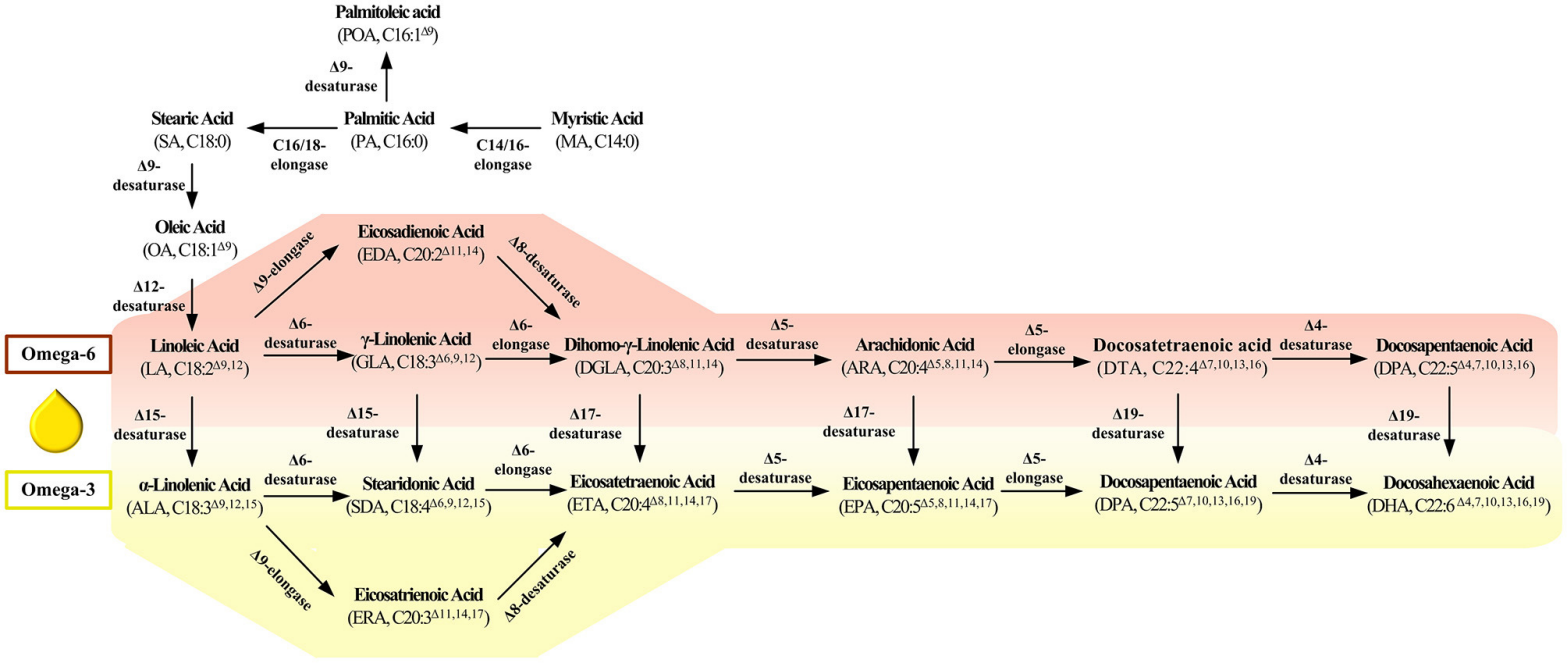
Key enzyme in the fatty acids' synthesis pathway.

Based on the previous studies, the synthesis approaches of LCPUFAs mainly consisted of the fatty acid synthesis pathway (FAS) and polyketosynthase (PKS) pathway (18, 19). Compared with PKS, the FAS pathway is more extensive and typical in all oleogenic microorganisms. As shown in Figure 1, many kinds of fatty acid desaturases and elongases play an essential role in synthesizing LCPUFAs, which perform the functions of introducing double bonds and extending the carbon chain. Thus, this paper reviews the features of and advances of these critical enzymes in LCPUFAs synthetic pathway. We also discuss the challenge and the most promising breakthrough direction of enzyme in LCPUFAs synthetic pathway, which aims to provide detailed information and novel ideas for the follow-up research in this field.

## Vital Desaturase in ω-6 and ω-3 Pathway

### Δ9-Desaturase

Δ9-desaturase is a central enzyme to synthesize the long chain monounsaturated fatty acids (LCMUFAs) from long chain saturated fatty acids (LCSFAs), which catalyze stearic acid (SA, 18:0) and palmitic acid (PA; C16:0) to oleic acid (OA, 18:1^Δ9^) and palmitoleic acid (POA, C16:1^Δ9^) (Figure 1). Scientists also have identified and characterized the various Δ9-fatty acid desaturase gene from *Rhodotorula toruloides* (20) and *Pseudomonas sp*. (21) (Table 1). These Δ9-desaturases shared three histidine-conserved boxes that would be the catalytic position of Δ9 fatty acid desaturase. The RtΔ9FAD protein was also predicted to have four possible transmembrane domains (54).

**Table 1:** The characteristic of the desaturase in ω-6 and ω-3 pathway.

**Desaturase**	**Source**	**Conversion rate**	**Gene (bp)**	**Amino acid**	**Molecular mass (kDa)**	**GeneBank No**.	**References**
Δ9	*Rhodotorula toruloides*	/	1,635	545	60.8	XP_016270987.1	(20)
	*Pseudomonas sp*.	/	1,182	394	45	AMX81567.1	(21)
	*Phaeodactylum tricornutum*	11.9%	1,227	408	46.36	/	(22, 23)
Δ12	*Isochrysis galbana*	/	1,158	386	42.8	ABD58898.1	(24)
	*Acanthamoeba castellanii*	/	1,224	407	/	ABK15557.1	(25)
	*Chlamydomonas sp*.	/	1,845	433	/	ACX42440.1	(26)
	*Calendula officinalis*	/	1,411	383	/	AAK26633.1	(27)
	*Helianthus annuus*	/	1,259	382	/	AAL68983.1	(28)
	*Chlorella vulgaris*	/	2,032	385	/	ACF98528.1	(29)
	*Phaeodactylum tricornutum*	/	1,526	436	/	AAO23564.1	(30)
	Haematococcus pluvialis	/	1,137	378	43.29	MH817076.1	(31)
	*Rhodotorula toruloides*	/	1,356	451	50.6	XM_016420199.1	(20, 32)
Δ6	*Glossomastix chrysoplasta*	7% to ALA, 6% to LA	1,821	465	51.9	AAU11445.1	(33)
	*Pythium splendens*	/	1,380	459	52.7	JX431892.1	(34)
	*Myrmecia incisa*	3.14% to LA, 2.21% to ALA	1,443	480	/	JN205756.1	(35)
	*Mucor rouxii*	/	1,831	467	/	AAR27297.1	(36)
	*Micromonas pusilla*	60% to ALA, 10% to LA	1,570	463	54.52	XM_003056946.1	(37)
	*Rhodococcus sp*.	/	1,242	413	45	AB847088.1	(38)
	*Pythium sp*.	62.7% to LA, 60.9% to ALA	1,401	466	52.8	ALE65995.1	(39)
	*Dunaliella salina*	/	1,329	422	/	/	(40)
	*Thalassiosira pseudonana*	/	1,455	484	/	AY817155.1	(41)
	*Isochrysis sp*.	2.3% to LA, 6.3% to ALA	1,478	482	~78	KR005946.1	(42)
	*Mucor sp*.		1,572	523		AB090360.1	(43)
Δ5	*Leishmania major*	5% to DGLA, 6% to ETA	1,254	417	/	HQ678521.1	(44)
	*Mortierella alpina*	12% to DGLA, 12.5% to ETA	1,341	446	/	GU593328.1	(44)
	*Ostreococcus tauri*	9% to DGLA, 11% to ETA	1,476	491	/	HQ678520.1	(44)
	*Ostreococcus lucimarinus*	6% to DGLA, 8% to ETA	1,476	491	/	HQ678519.1	(44)
	*Paramecium tetraurelia*	13% to DGLA, 14% to ETA	1,542	513	/	HQ678517.1	(44)
	*Oblongichytrium sp*.	24.8% to DGLA, 36.6% to ETA	1,308	435	50	AB432913.1	(45)
	*Thraustochytrium sp*.	19.9% to DGLA, 22.9% to ETA	1,320	439	/	EU643618.1	(46, 47)
	*Isochrysis sp*	/	1,170	382	70	KR062001.1	(48)
Δ4	*Isochrysis galbana*	34 % to DPA	1,302	433	48.1	JQ664598.1	(49)
	*Isochrysis sphaerica*	79.8 to DPA	1,284	427	47.9	JQ791105.1	(50)
	*Ostreococcus lucimarinus*	10% to DPA, 4% to DTA	1,409	459	/	XM_001415706.1	(51)
	*Pavlova lutheri*	~30% to DPA and DTA	1,619	445	49	AAQ98793.1	(52)
	*Pavlova viridis*	/	1,440	479	52.7	GU594191.1	(53)

As the obstacles in harvest crystal structures of fatty acid desaturases located in the membrane, homology modeling could predict the three-dimensional structures. The docking studies could be employed to predict the active center of the desaturases. Predicted by docking results, His34, His71, and, His206 were the possible residues to contact with the docked palmitic acid, located in the first, second, and third conserved histidine boxes (21). Another study of homology modeling and docking also showed that four α-helices constitute the catalytic site and transmembrane domain in Δ9-desaturase from *Arthrospira platensis* (55). Beyond that, 3D structure modeling studies revealed that three variant amino acids (F160, A223, and L156) in domain Δ9-desaturase encoded by gene *PtAAD* narrow the space of substrate binding center, explaining the reason of substrate preference of this especial Δ9-desaturase for palmitic acid (22, 23) (Table 2).

**Table 2:** The effect of site directed mutagenesis on the performance of desaturase.

**Enzyme**	**Source**	**Mutation site**	**Performance**	**References**
Δ9-desaturase	*Phaeodactylum tricornutum*	F160L, A223T, and L156M	Substrate preference changes from C16:0 to C18:0	(23)
Δ12-desaturase	*Mortierella alpina*	P166L or H116Y	Loss the catalytic activity from 18:1^Δ^9 to 18:2^Δ9,12^	(56)
Δ12-desaturase	*Mortierella alpina*	W131L or S218G or N389D	Activity weakened slightly	(56)
Δ6-desaturase	*Micromonas pusilla*	F419V or A374Q	Activity decreases to the half of wild type	(37)
		Q409R or M242P	Completely inactivated	(37)
		Q236N or A423C	Activity enhanced slightly	(37)
		Q190A, S197Q, and Q209G	No significant change	(37)
		V399I/I400E, E222S, and M227K	Activity decreases to 40.26, 31.42, and 31.61%, respectively (wild type: 71.37%).	(57)
		G194L	Activity decreases to 6.5%, respectively (wild type: 71.37%).	(57)
Δ15-desaturase	*Chlamydomonasreinhardtii*	T286S	No significant change	(1)
		T286Y, T286H, T286C, or T286G	Loss of catalytic activity	(1)
	*Mortierella alpina*	E111D, T322S, and F353H	No significant change to wild type	(58)
		W106F and V137T	Markedly decreased the conversion rate for AA (40 to 50%)	(58)
		A44S, M156I, and W291M	Markedly increase the conversion rate for AA (30–40%)	(58)

By overexpressing the *Rt*Δ*9FAD* gene into *R. toruloides*, the transformant produces 5-fold more oleic acid content in total amount (54). At the same time, Liu and his colleague (23) reported a Δ9-desaturase from *Phaeodactylum tricornutum* to prefer palmitic acid-ACP as a substrate to promote the palmitoleic acid under average and stress culture conditions. Rau and his colleague also find that the Δ9-desaturase from *Aurantiochytrium sp*. T66 could accept palmitic acid and stearic acid as substrates (59). The reasonable explanation for substrate preference was also given out through homology modeling and docking in the last paragraph.

### Δ12-Desaturase

Δ12-desaturase is a vital enzyme in the first step of LCPUFAs synthesized from LCMUFAs, which catalyze oleic acid (OA, 18:1^Δ9^) to linoleic acid (LA, 18:2^Δ9,12^) (Figure 1). Scientists also have identified and characterized the various Δ12-fatty acid desaturase gene from *Isochrysis galbana* (24), *Acanthamoeba castellanii* (25), *Chlamydomonas sp*. (26), *Calendula officinalis* (27), *Helianthus annuus* (28), *Chlorella vulgaris* (29), *P. tricornutum* (30), and *Haematococcus pluvialis* (31), whose detailed information is listed in Table 1. These Δ12-desaturases also share three conserved histidine boxes, which are considered the catalytic center and are critical for desaturase activity.

Different environment stressors will influence the *FAD2* (coding Δ12-desaturase) transcription of the oleaginous microorganism. In *Y. lipolytica*, the low temperature or substrate (n-alkanes or oleic acid) induce the upregulation of Δ12-desaturase (60). Similarly, low temperature (15°C), high salinity (salinity of 62 and 93%), and nitrogen starvation (220 μmol/L) upregulate the abundance of *IgFAD2* transcript in *I. galbana* as well (24).

Overexpress Δ12-desaturase in oleogenic microorganisms could enhance the product accumulation significantly. After heterologous overexpress Δ12-desaturase from *M. alpina* or *Fusarium verticillioides* in the oleaginous yeast *Rhodosporidium toruloides*, linoleic acid concentration increased up to 1.3 g/L, which was 5-fold higher than that in the parent strain (61). Overexpression of endogenous *RtFAD2* in *R. toruloides* also improved lipid and linoleic acid (32). Conversely, the growth rate was slower at 12°C under the deletion of *FAD2* gene (coding Δ12-desaturase) in *Y. lipolytica*, which was recovered by the addition of 18:2, not 18:1.

### Δ6-Desaturase

Δ6-desaturase catalyze linoleic acid (LA, C18:2^Δ9,12^) and α-linolenic acid (ALA, C18:3^Δ9,12,15^) to γ-linolenic acid (GLA, C18:3^Δ6,9,12^) and produce stearidonic acid (SDA, C18:4^Δ6,9,12,15^). Scientists also have identified and characterized the various Δ6-desaturase from *Glossomastix chrysoplasta* (33), *Pythium sp*. (39), *Isochrysis* sp. (42), and *Mucor sp*. (43). Araki and his colleague identified a novel gene encoding Δ6- desaturase from *Rhodococcus sp*. Different from others, this desaturase preferred saturated fatty acids as substrates and catalyzed hexadecanoic acid to cis-6-hexadecenoic acid (38) (Table 1).

By analyzing the sequence of the Δ6-desaturase from *Mucor sp*., Jiang et al. found that −919 to −784 bp in the promoter region plays a vital role in the high activity of Δ6-desaturase (62). Compared with native Δ6-desaturase in *Pythium sp*., the codon-optimized strategy could markedly enhance Δ-6 desaturated products, in which the substrate conversion rates of LA and ALA increased from 5.4 and 4.2% to 62.7 and 60.9%, respectively (39). Zhu et al. (63) found that overexpression of endogenous Δ6-desaturase significantly enhances the eicosapentaenoic acid accumulation in *P. tricornutum*, compared with overexpression of heterologous one (64). However, the other scientist reported another result that differed massively in *M. alpina*. First, the Δ-6 desaturase from *M. alpina* (57) and *M. pusilla* (65) has the characteristic of significant substrate preference, in which the *MpFADS6* (from *M. pusilla*) and *MaFADS6-I* (from *M. alpina*) prefer to ALA and LA, respectively. Zhang et al. (34) also isolated and identified a Δ6-desaturase from *Pythium splendens* with a preference to LA. Second, they further introduced the exogenous gene encoding ALA-preferring Δ6-desaturase from *M. pusilla* into the *M. alpina*, EPA yield was increased from 22.99 ± 2.7 mg/L in WT *M. alpina* up to 588.5 ± 29.6 mg/L in engineered one in 5-L fermentation, in which peony seed oil (0.1%) and peony seed meal (50 g/L) were exogenously added as a substitution to ALA (66). Last, they introduced the exogenous gene from *Thalassiosira pseudonana* (41), encoding a higher Δ6-desaturase activity (*TpFADS6*) for ALA, to the high ALA producer of *Dunaliella salina* (40). After performing culture conditions optimization, the EPA concentration increased from 1.6 ± 0.2 to 554.3 ± 95.6 mg/L, 343.8-fold higher than that in the wild-type strain (40). Beyond that, the expression of *IsFAD6* (encoding Δ6-desaturase) was upregulated in high salinity, low temperature, and high nitrogen deficiency culture condition, indicating *IsFAD6* respond to the various abiotic stresses (61). At the same time, heterologous expression Δ6-desaturase in *Nannochloropsis oceanica* enhanced both growth and photosynthetic efficiency (67).

Scientists also focused on studying detailed characteristics to harvest a deeper insight into the mechanism. Song et al. (68) demonstrated that amino acid residues 114–174, 206–257, and 258–276 play a vital role in substrate recognition for Δ6-desaturase. Shi et al. (57) also found that MpFADS6 (from *M. pusilla*) and MaFADS6-I (from *M. alpina*) showed a difference in substrate preference. Further studies based on the domain swapping approach reveal that sequences between the histidine boxes I and II played a pivotal role in which mutation of G194, E222, M227, and V399/I400 cause a significant decrease in the ALA conversion rate of MpFADS6 (57) (Table 2). By employing site-directed mutation, the scientist found that mutants Q409R and M242P lost the desaturation function, while mutants F419V and A374Q weakened the catalytic activities. Combined with molecular modeling, they reveal that electronic transfer in the catalytic process correlated with histidine-conserved region III, while desaturation is highly correlated with histidine-conserved regions I and II (37) (Table 2). These results from site-specific mutagenesis and molecular modeling bridge the gap between structure and the catalytic mechanism of these desaturases.

### Δ5-Desaturase

Δ5-desaturase catalyze dihomo-γ-linolenic acid (DGLA, C20:3^Δ8,11,14^) and eicosatetraenoic acid (ETA, C20:4^Δ8,11,14,17^) to arachidonic acid (AA, C20:4^Δ5,8,11,14^) and eicosapentaenoic acid (EPA, C20:4^Δ5,8,11,14,17^). Scientists have also identified and characterized the various Δ5-desaturase genes from *Paramecium tetraurelia* (44), *Ostreococcus tauri* (44), *Ostreococcus lucimarinus* (44), *Thraustochytrium sp*. (46), and *Oblongichytrium sp*. (45). The amino acid sequence from this Δ5-desaturase was significantly homologous, containing three conserved histidine boxes and a cytochrome b5 domain (Table 1).

Tavares et al. (44) carried out a research on substrate preferences and desaturation efficiencies of Δ5-desaturase from *P. tetraurelia, O. tauri*, and *O. lucimarinus*. Their results also demonstrated that Δ5-desaturase from *O. tauri, O. lucimarinus, M. alpina*, and *P*. *tetraurelia* prefer the substrates bound in phospholipid to a promiscuous acyl carrier substrate, while Δ5-desaturase from *L. major* was an acyl coenzyme A-dependent.

Heterologous expression Δ5-desaturase from *Thraustochytrium aureum* in *Aurantiochytrium limacinum* triggers increase of AA and EPA by 4.6- and 13.2-fold, which is driven by the thraustochytrid ubiquitin promoter (47). After disrupting the Δ5-desaturase in *M. alpina*, scientists achieve a higher percentage of DGLA (40.1%) accumulation in total lipid (69). At the same time, overexpressing the Δ5-desaturase in *P. tricornutum* exhibited a significant increment of unsaturated fatty acids, EPA (increase by 58%), and neutral lipid content (increase up to 65%) (70). Thus, these results demonstrated the critical role of Δ5-desaturase in catalyzing the DGLA and ETA to AA and EPA, respectively.

### Δ4-Desaturase

Δ4-desaturase catalyze docosatetraenoic acid (DTA, C22:4^Δ7,10,13,16^) and docosapentaenoic acid (DPA, C22:5^Δ7,10,13,16,19^) to docosapentaenoic acid (DPA, C22:5^Δ4,7,10,13,16^) and docosahexaenoic acid (DHA, C22:6^Δ4,7,10,13,16,19^). As very important LCPUFAs, more researchers focus on another more efficient PKS pathway to synthesize DHA from *Schizochytrium sp*. However, the scientist also identified this desaturase encoding gene from *I. galbana* (49), *Isochrysis sphaerica* (50), *Pavlova lutheri* (52), *and Pavlova viridis* (53). Among them, the one from *P. lutheri* desaturated DTA (C22:4^Δ7,10,13,16^) and DPA, (C22:5^Δ7,10,13,16,19^) (52), while the others from *I. galbana* and *I. sphaerica* prefer DPA (C22:5^Δ7,10,13,16,19^) as substrate (49, 50) (Table 1).

## Catalyze Performance of ω3-Desaturase

### Δ15-Desaturase

Some desaturase can convert ω-6 fatty acids to ω-3 fatty acids, which is named ω-3 desaturase. Δ15-desaturase is a kind of ω3-desaturase with C18 fatty acid as substrate, which catalyzes linoleic acid (LA, 18:2^Δ9,12^) and γ-linolenic acid (GLA, C18:3^Δ6,9,12^) to α-linolenic acid (ALA, C18:3^Δ9,12,15^) and stearidonic acid (SDA, C18:4^Δ6,9,12,15^), respectively. Scientists found that low temperature and high salinity could motivate the upregulation of *CiFAD3* (coding Δ15-desaturase) expression in *Chlamydomonas reinhardtii* (71).

Substrate preference was an exciting topic determined by the structure and amino acid in the enzyme's binding site. Scientists found that Δ15-desaturase from *Riftia pachyptila* (72) and *M. alpina* (73) show a preference for C18 fatty acids, while Δ17-desaturase from *Pythium aphanidermatum* (74) display a higher catalytic activity for C20 fatty acids (Table 3). On combining site directed mutagenesis, homology modeling, and molecular docking, scientists revealed that the W106 and V137 related to substrate recognition (mutations in these amino acids significantly decreased the enzyme activity), and the A44, M156, and W291 residues related to the higher desaturation activity for C20 substrates (mutations in these amino acids markedly increase the conversion rate of AA). Beyond that, the amino acids residues that bind to CoA groups govern substrate preference (58) (Table 2). Scientists also found that the threonine residue located in the fourth transmembrane was essential for the typical structure and function of Δ15-desaturase in *C. reinhardtii*, and the mutations in this site resulted in varying degrees of activity weaken (1) (Table 2).

**Table 3:** The characteristic of the ω3-desaturase.

**Desaturase**	**Source**	**Conversion rate (%)**	**Gene (bp)**	**Amino acid**	**Molecular Mass (kDa)**	**GeneBank No**.	**References**
Δ15	*Chlamydomonas sp*.	/	1,845	433	49.2	GQ888689.1	(26)
	*Mortierella alpina*	59.7% to LA, 29.6% to AA	1,212	403		KF433065.1	(73, 75)
	*Riftia pachyptila*	3.4% to LA, 4.2% to GLA	1,587	403	/	KY399781.1	(72)
Δ17	*Pythium aphanidermatum*	63.8% to AA	1,533	/	/	FW362186.1	(74)
	*Phytophthora sojae*	60% to AA	1,092	/	/	FW362213.1	(74)
	*Phytophthora ramorum*	65% to AA	1,086	/	/	FW362214.1	(74)

### Δ17-Desaturase

Δ17-desaturase is another kind of ω3-desaturase with C20 fatty acid as a substrate which catalyzes dihomo-γ-linolenic acid (DGLA, C20:3^Δ8,11,14^) and arachidonic acid (AA, C20:4^Δ5,8,11,14^) to eicosatetraenoic acid (ETA, C20:4^Δ8,11,14,17^) and eicosapentaenoic acid (EPA, C20:4^Δ5,8,11,14,17^), respectively. Considerable efforts have been focused on identified and characteristic Δ17-desaturase, which could convert 20°C ω-6 fatty acids to ω-3 fatty acids. Scientists have identified the Δ17-desaturase from *P. aphanidermatum* (74), *Phytophthora sojae* (74), *Phytophthora ramorum* (74), *Saprolegnia diclina* (76), and *Phytophthora infestans* (77) (Table 3). Among them, Δ17-desaturase from *S. diclina, P. aphanidermatum, P. sojae*, and *Phytophthora ramorum* exhibited a great preference to convert AA to EPA. Thus, Ge and his colleague (78) transformed the Δ17-desaturase encoding gene from *P. aphanidermatum* into *M. alpina* to achieve EPA production with a 49.7% conversion rate of AA. Tang and his colleague identified a new Δ17-desaturase from *Phytophthora parasitica*, exhibiting high activity for C20 substrate (conversion rate was 70% for AA) and week activity for C18 substrate. They further introduce the gene *PPD17* encoding this Δ17-desaturase into the *M. alpina*, resulting in the conversion of AA to EPA (1.9 g/L) (79). In our previous work, a Δ17-desaturase encoding gene from *S. diclina* was also introduced into the genome of the *Schizochytrium* sp. through homologous recombination. Compared with the wild-type strains, the ω-3/ω-6 ratio in fatty acid in genetically modified strains increased from 2.1 to 2.58, and 3% of DPA was converted to DHA (80).

## Characteristic of Fatty Acid Elongase

### Δ6-Elongase

Δ6-elongase is a kind of fatty acid elongase with C20 fatty acid as a substrate which catalyze γ-linolenic acid (GLA, C18:3^Δ6,9,12^) and stearidonic acid (SDA, C18:4^Δ6,9,12,15^) to dihomo-γ-linolenic acid (DGLA, C20:3^Δ8,11,14^) and eicosatetraenoic acid (ETA, C20:4^Δ8,11,14,17^), respectively. Yu et al. (81) identified a Δ6-elongase localized to the endoplasmic reticulum, whose expression level was enhanced by nitrogen starvation. Jeennor et al. (82) identified a Δ6-elongase gene from *Pythium* sp., exhibiting a high specificity for C18 PUFAs with a double bond at Δ6-position. Co-overexpression of Δ9-desaturase, Δ12-desaturase, and Δ6-elongase in *Aspergillus oryzae*, scientists achieve success in enhancing free dihomo-γ-linolenic acid production with a yield of 284 mg/L (83). Shi et al. (84) identified a Δ6-elongase *N. oceanica*, which applied its elongated function on C18 PUFAs with a double bond at Δ6-position. This elongase encoding gene not only attenuated DGLA, ARA, and EPA content, but also enhanced GLA content, supporting the vital role of this enzyme in the exclusive ω-6 pathway of EPA biosynthesis (84) (Table 4).

**Table 4:** The characteristic of the elongase.

**Elongase**	**Source**	**Conversion rate (%)**	**Gene (bp)**	**Amino acid**	**Molecular Mass (kDa)**	**GeneBank No**.	**References**
Δ6	*Myrmecia incisa*	24 to GLA 41 to SDA	1,331	288	29.9	EU846098.1	(81)
	*Pythium sp*.	29.3 to GLA 36.5 to SDA	837	277	32.1	KJ546459.1	(82)
	*Nannochloropsis oceanica*	70.5 to GLA 34.6 to SDA	831	276	/	KY214452.1	(84, 85)
Δ5	*Pavlova salina*	30.2 to EPA	1,220	302	/	AY926605.1	(86)
	*Pavlova viridis*	/	1,228	314	34	EF486525.1	(87)
	*Phaeodactylum tricornutum*	87.9 to EPA	1,110	369	/	XP_002176686.1	(88)

### Δ5-Elongase

Δ5-elongase is a kind of fatty acid elongase with C20 fatty acid as substrate which catalyze arachidonic acid (AA, C20:4^Δ5,8,11,14^) and eicosapentaenoic acid (EPA, C20:4^Δ5,8,11,14,17^) to Docosatetraenoic acid (DTA, C22:4^Δ7,10,13,16^) and Docosapentaenoic acid (DPA, C22:5^Δ7,10,13,16,19^), respectively. Employing fluorescent protein as an indicator, Niu et al. (87) found the Δ5-elongase from *Pavlova viridis* located in the endoplasmic reticulum. Robert and his colleague identified a Δ-5 elongase from *Pavlova salina* and characterized the exclusive elongase function for EPA. Furthermore, the scientist heterologous expressed the gene encoding Δ5-elongase in the moss *Physcomitrella patens* from the algae *Pavlova sp*. and harvested 4.51 mg/l Docosapentaenoic acid (DPA, C22:5^Δ7,10,13,16,19^) from endogenous arachidonic acid (89). Jiang et al. identified a Δ5-elongase from *P. tricornutum*, exhibiting a substrate preference for EPA. Co-expressed the Δ5-elongase and Δ4-desaturase in *Pichia pastoris*, they successfully construct a pathway for docosahexaenoic acid biosynthesis (88) (Table 4). There also exists another alternative pathway for Δ6-elongase/Δ6-desaturase, which is called Δ9-elongase/Δ8-desaturase pathway originated from euglenoid species (74). Δ9-elongase is a kind of fatty acid elongase with C18 fatty acid as a substrate which catalyze linoleic acid (LA, C18:2^Δ9,12^) and α-linolenic acid (ALA, C18:3^Δ9,12,15^) to eicosadienoic acid (EDA, 20:2 C18:3^Δ11,14^) and eicosatrienoic acid (ERA, C20:4^Δ11,14,17^), respectively. Then, Δ8-desaturase further catalyze EDA and ERA to dihomo-γ-linolenic acid (DGLA, C20:3^Δ8,11,14^) and eicosatetraenoic acid (ETA, C20:4^Δ8,11,14,17^). However, compared with other elongase and desaturase, there is relatively little research about these enzymes.

## Enzyme Structure Analysis

### Structure Analysis of Front-End Desaturase

The desaturases, introduce a double-bound between the carboxyl (end) and the original double bonds (front) in the substrate, were defined as front-end desaturase, including Δ4, Δ5, Δ6, Δ9, and Δ12 desaturase. Thus, these enzymes share similar characteristics in a structure. First, these enzymes consisted of hydrophobic transmembrane regions and hydrophilic nontransmembrane regions. However, they still exhibit differences in evolution. For example, the number of the transmembrane regions was different in each enzyme, in which the number is 2, 3, and 4 for Δ4, Δ6, and Δ8 desaturase, respectively. Second, these enzymes are fellowed by cytochrome b5-like binding domain with “HPGG” motif in the N-terminus, which is reserved for cytochrome b5 and NAD(P)H as electron donors (31, 51). Third, nontransmembrane regions contain three histidine-rich motifs, which were considered for the binding position of di-iron and are critical for desaturase activity (21, 31). The three histidine-rich motifs that were highly conservative consisted of HXXXH, HXXHH, and (H/Q)XXHH in this enzyme, which demonstrated the similarity and homology of them in origin. Thus, the mutation of the amino acid close to the substrate-binding region (three histidine-rich motifs) will cause a significant impact on desaturase activity and substrate preference. As shown in Table 2, the mutations of these functional domains (F160L, A223T, and L156M), located at the bottom of substrate binding position, cause the substrate preference changes from C16:0 to C18:0 (23).

### Structure Analysis of ω3-Desaturase

The structure of ω3-desaturase was similar to that of front-end desaturase, which consisted of hydrophobic transmembrane regions and hydrophilic nontransmembrane regions (79). In detail, ω3-desaturase included three highly conservative histidine-rich motifs (HXXXH, HXXHH, and (H/Q)XXHH) and more than four transmembrane domains (1, 26). The three highly conservative histidine-rich motifs were also predicted as the critical position of di-iron for substrate binding. However, cytb5 “HPGG” motif was not located in the N terminal of the enzyme. Beyond that, many amino acids were conservative in this ω3-desaturase. As shown in Table 2, T286 in Δ15-desaturase from *Chlamydomonas reinhardtii* was responsible for catalytic activity (1) while A44S, W106, E111, M156, V137, W291M, T322S, and F353H in Δ15-desaturase in Δ15-desaturase from *Mortierella alpina* was responsible for substrate preference (58).

### Structure Analysis of Elongase

The characteristic of amino acid sequence exhibit a significant difference with that of desaturase, which may also lead to the difference in structure. In detail, elongase contains seven transmembrane regions and three/five different kinds of conservative motifs. The motifs in Δ5-elongase consisted of a histidine-rich box (SFLHVYHHV), a tyrosine-rich box (YLTQAQLVQF), and a conserved motif (MYXYY) in (87). However, Δ6-elongase contains four motifs (KxxExxDT, QxxFLHxYHH, NxxxHxxMYxYY, and TxxQxxQ) and a histidine-rich catalytic motif (and HVYHH) (82). These conservative motifs were essential for di-iron binding and responsible for enzyme activity.

### Structure Analysis Method

**Figure 2:**
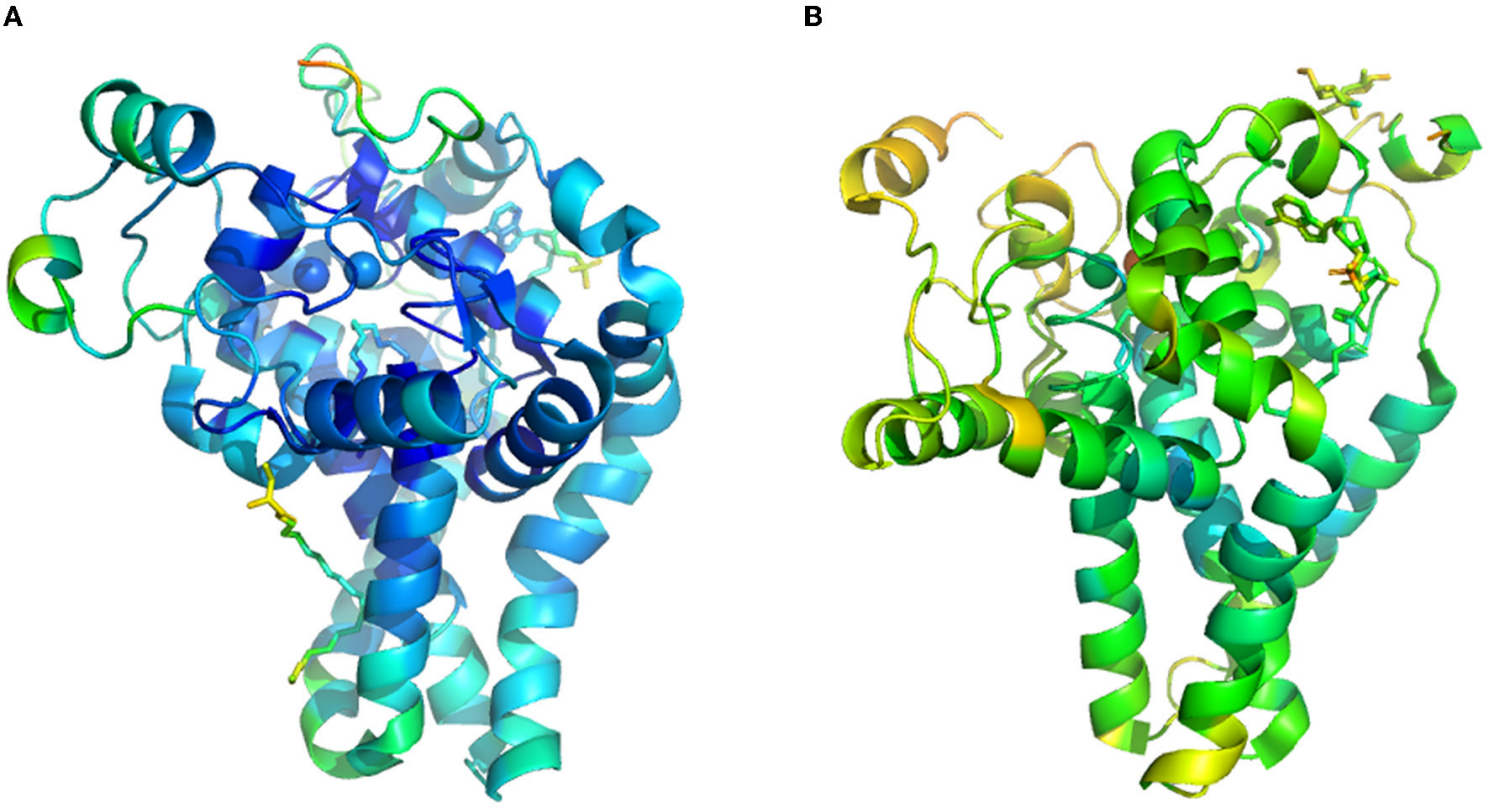
The crystal structure of transmembrane fatty acid desaturases. **(A)**, crystal structure of mouse stearoyl-CoA desaturase (PDB ID: 4YMK). **(B)**, human integral membrane stearoyl-CoA desaturase (PDB ID: 4ZYO).

Enzyme structure model and information are collected from protein crystals, which is hindered by a lack of time and labor resources. These difficulties are especially marked in transmembrane protein, including fatty acid desaturase and elongase, widely distributed in cytomembrane, endoplasmic reticulum, and chloroplast membrane. Currently, only two kinds of three-dimensional structures of the membrane fatty acid desaturases are available, one is the mouse stearoyl-CoA desaturase (Figure 2A, PDB ID: 4YMK), and the other is human integral membrane stearoyl-CoA desaturase (Figure 2B, PDB ID: 4ZYO). Thus, homology modeling and docking are the essential methods to analyze the structure of bacterial membrane fatty acid desaturases and elongase in the current state (21). Herein, we summarized the research approach of homology modeling and docking based on the previous work (21–23, 55). First, after homology analyzes the amino acid sequence, the scientist can determine templates with higher homology and resolution in the protein database (e.g., Protein Data Bank, PDB). Second, scientists began to predict the structure based on the crystal structure of templates by employing some structure prediction software (e.g., Swiss Model and Modeler). Third, a scientist will assess the structure from many predictions. After score and energy minimization, some software will select the optimal structural model from the candidate. Fourth, the active site of the structural model was predicted using the software. Last, the docking simulations process was performed by the software of Autodock. The scientist could run the docking result through Gromacs and harvest stable conformation information with a substrate binding in the active site, which was then evaluated using RMSD (Root Mean Squared Deviation). Combined with site-specific mutagenesis, a scientist could match the performance of the mutant with structure variation, which could provide a reasonable explanation for the mutation (22, 58).

## Conclusions and Perspectives

Dietary bioactive lipids are important nutriments to maintain the typical metabolic status of the organism. As the practical component in lipid, the crucial role is self-evident of LCPUFAs in boosting the development of the brain and immune system and preventing cardiovascular disease. More importantly, what chip is to the electronic equipment, an enzyme is to the cell factory. These fatty acid synthesis related enzymes play a vital role in the desaturation and elongation of the carbon chain. These enzymes exist widely in plants, fungus, microalgae, and bacteria located in the cell and/or organelle membrane, including endoplasmic reticulum and chloroplast. Thus, the Open Reading Frames and amino acid sequence of these enzymes share a high degree of homology among the genera, which also provide evidence for the origins and evolutionary processes. Employing site-specific mutagenesis, homology modeling, and docking studies, scientists reveal that the structure made by the amino acids at a specific site contributes to substrate preference and catalytic activity. Many enzyme engineering strategies used for high content LCPUFAs rich oil synthesis were also summarized. Thus, the review of these important advances on desaturase and elongase could not only provide extensive data for enzyme rational design and modification but also light up the way for the efficiency LCPUFAs rich oil production.

As shown in Table 2, many researchers have created the desaturase mutants with single-site and multisite. Combining the catalytic performance of each mutant with the results from homologous modeling, they can deduce the position of crucial amino acids related to the catalytic activity and substrate preference. However, it is a nonrational and time-consuming process that much work was an indispensable part of harvesting positive mutants. Moreover, most of the time, we do not obtain positive mutants, fortunately. So, it is essential to utilize these hard-earned data, whether positive or negative. Thus, the database could be constructed from positive and negative catalytic performance as listed in Table 2, which could serve as a sample of artificial intelligence (AI) learning. After training and learning are repeated, it will obtain the undisclosed objective laws and provide us a better experimental scheme potential. At last, the AI model will guide us in moving in the right direction and approach the enzyme with higher catalytic activity quickly (90, 91).

Scientists have identified many kinds of desaturase and elongase from different organisms and analyzed their amino acid composition and substrate preference. However, the lacke of three-dimensional crystal structure data still restricts the comprehensive and deep analysis of these enzymes, which remain an excellent challenge for researcher. On the one hand, the cryo-electron microscopy technique has been widely applied in analyzing transmembrane protein, which could provide a reliable experimental basis for modeling (92). On the other hand, the rapid-developed AI techniques (such as AlphaFold and RoseTTAFold) are also a powerful tool for protein structure prediction, which could guide enzyme design and modifications (93, 94). Combined crystal structure analysis with molecular dynamics simulation, we can explore more information from catalytic efficiency, structure change, electron and proton transfer, which could provide a rational strategy to enhance catalytic performance, change substrate preference, improve catalytic stability. Novel technology, including nanopore (95), scanning tunneling microscope-break junction (96), atomic force microscope (97), and optical tweezers (98) will enable scientists to go a step further in enzyme research. Beyond that, metabolic engineering equipped with machine learning techniques (neural network and Bayesian optimization etc.) (99, 100), and omics analysis (101, 102) could also be employed to design, regulate, and optimize the metabolic pathway for high-efficiency LCPUFAs rich oil production. In the future, comprehensive interdisciplinary research will become the theme and contribute to enzymatic research.

## Author Contributions

X-YZ, Y-HZ, A-FX, and B-SF planned the manuscript, wrote and revised it. They were helped by A-HZ in revision and writing. All authors contributed to the article and approved the submitted version.

## Funding

The National Postdoctoral Program for Innovative Talents (No. BX20200197) and National Natural Science Foundation of China (No. 21978245).

## Conflict of Interest

The authors declare that the research was conducted in the absence of any commercial or financial relationships that could be construed as a potential conflict of interest.

## Publisher's Note

All claims expressed in this article are solely those of the authors and do not necessarily represent those of their affiliated organizations, or those of the publisher, the editors and the reviewers. Any product that may be evaluated in this article, or claim that may be made by its manufacturer, is not guaranteed or endorsed by the publisher.
