# Corrigendum: Key Enzymes in Fatty Acid Synthesis Pathway for Bioactive Lipids Biosynthesis

**DOI:** 10.3389/fnut.2022.914273

**Published:** 2022-04-25

**Authors:** Xiao-Yan Zhuang, Yong-Hui Zhang, An-Feng Xiao, Ai-Hui Zhang, Bai-Shan Fang

**Affiliations:** ^1^College of Food and Biological Engineering, Jimei University, Xiamen, China; ^2^Department of Chemical and Biochemical Engineering, College of Chemistry and Chemical Engineering, Xiamen University, Xiamen, China

**Keywords:** bioactive lipids, desaturase, elongase, fatty acid synthesis pathway, oleogenic microorganisms

In the published article, there was an error in “Affiliation 1.” Instead of “Department of Chemical and Biochemical Engineering, College of Chemistry and Chemical Engineering, Jimei University, Xiamen, China,” it should be “College of Food and Biological Engineering, Jimei University, Xiamen, China.”

The authors apologize for this error and state that this does not change the scientific conclusions of the article in any way. The original article has been updated.

## Publisher's Note

All claims expressed in this article are solely those of the authors and do not necessarily represent those of their affiliated organizations, or those of the publisher, the editors and the reviewers. Any product that may be evaluated in this article, or claim that may be made by its manufacturer, is not guaranteed or endorsed by the publisher.

